# Preparation of TiO_2_/SnO_2_ Electron Transport Layer for Performance Enhancement of All-Inorganic Perovskite Solar Cells Using Electron Beam Evaporation at Low Temperature

**DOI:** 10.3390/mi14081549

**Published:** 2023-08-01

**Authors:** Tao Xue, Ting Li, Dandan Chen, Xiao Wang, Kunping Guo, Qiang Wang, Fanghui Zhang

**Affiliations:** School of Electronic Information and Artificial Intelligence, Shaanxi University of Science and Technology, Xi’an 710021, China

**Keywords:** perovskite solar cells, bilayer electron transfer layer, electron beam evaporation

## Abstract

SnO_2_ has attracted much attention due to its low-temperature synthesis (ca. 140 °C), high electron mobility, and low-cost manufacturing. However, lattice mismatch and oxygen vacancies at the SnO_2_/CsPbI_3−x_Br_x_ interface generally lead to undesirable nonradiative recombination in optoelectronic devices. The traditional TiO_2_ used as the electron transport layer (ETL) for all-inorganic perovskite solar cells (PSCs) requires high-temperature sintering and crystallization, which are not suitable for the promising flexible PSCs and tandem solar cells, raising concerns about surface defects and device uniformity. To address these challenges, we present a bilayer ETL consisting of a SnO_2_ layer using electron beam evaporation and a TiO_2_ layer through the hydrothermal method, resulting in an enhanced performance of the perovskite solar cell. The bilayer device exhibits an improved power conversion efficiency of 11.48% compared to the single-layer device (8.09%). The average fill factor of the bilayer electron transport layer is approximately 15% higher compared to the single-layer electron transport layer. Through a systematic investigation of the use of ETL for CsPb_3−x_Br_x_ PSCs on optical and electronic properties, we demonstrate that the SnO_2_/TiO_2_ is an efficient bilayer ETL for PSCs as it significantly enhances the charge extraction capability, suppresses carrier recombination at the ETL/perovskite interface, facilitates efficient photogenerated carrier separation and transport, and provides high current density and reduced hysteresis.

## 1. Introduction

Perovskite solar cells (PSCs) have garnered significant attention due to their straightforward fabrication process, high carrier mobility, long carrier diffusion length, and strong optical absorption coefficient [1,2,3,4,5,6]. These perovskite materials are widely recognized as one of the most promising next-generation solar materials [7,8,9,10,11]. The remarkable progress in organic–inorganic hybrid perovskite solar cells is particularly noteworthy, with the power conversion efficiency (PCE) rising from 3.8% to 25.7% [12,13,14,15] within a decade. However, the organic component in perovskite materials poses challenges as it is prone to decomposition and instability in high humidity and oxygen-rich environments. Thermal stability is also a concern, as organic ions can lead to decomposition under conditions of high humidity and temperature [16,17,18,19]. Conventional device structures for organic–inorganic perovskite solar cells generally rely on expensive metal electrodes and organic hole transport layers (HTLs), which can limit commercial production to some extent [20,21,22]. In the pursuit of perovskite solar cells with improved stability, excellent photoelectric properties, a low cost, and no toxicity, researchers have turned their attention to carbon-based all-inorganic perovskite solar cells. This emerging field has gained considerable momentum, and over the past few years, the PCE of carbon-based all-inorganic perovskite solar cells has shown steady growth [23,24,25].

The electron transport layer (ETL) plays a crucial role in the performance of perovskite solar cells (PSCs). Its film flatness directly impacts the perovskite film’s performance by blocking holes, transporting electrons, and reducing recombination within PSCs. Titanium dioxide (TiO_2_) is widely used as an ETL in PSCs due to its optical transparency and chemical stability. However, its low electron mobility and extraction ability lead to charge accumulation at the ETL/perovskite interface, resulting in reduced efficiency and stability. Typically, TiO_2_ is prepared using spray pyrolysis or sol-gel methods, followed by a high-temperature sintering step (>450 °C) that is crucial for the crystallization and improved electrical conductivity of the TiO_2_ layer [26]. Nevertheless, high-temperature processes are incompatible with flexible substrates and monolithic in-line systems.

To overcome these limitations, tin dioxide (SnO_2_) has emerged as a popular ETL alternative. SnO_2_ offers advantages such as a low synthesis temperature (around 140 °C), wide bandgap, high transmittance, high carrier mobility, good chemical stability, and low manufacturing cost [27,28,29]. In 2015, Fang et al. [30] first applied SnO_2_ as an ETL in perovskite solar cells. Subsequently, Jiang, Q et al. [31] reported the development of high-efficiency perovskite solar cells using SnO_2_ as an ETL, benefiting from lower energy levels at the bottom of the conduction band (ECBM), facilitating electron transfer and reducing charge accumulation at the interface. Yet, lattice mismatches and oxygen vacancies at the ETL/perovskite interface significantly affect the performance of SnO_2_-based perovskite solar cells [31,32,33]. These cells exhibit relatively low electroluminescent external quantum efficiency (EQE), resulting in higher open-circuit voltage (*V*_oc_) losses due to the lower ECBM [34]. It is evident that a single-layer ETL with a specific ECBM favors electron transport but decreases *V*_oc_, and vice versa. A high-quality electron transport layer (ETL) is of paramount importance in device fabrication and plays a crucial role in achieving high-efficiency perovskite solar cells. Ensuring efficient charge extraction and transport within the ETL is essential for enhancing the overall performance of the solar cell. However, monolayer ETLs often encounter challenges such as lattice mismatches, oxygen vacancies, and energy level misalignment at the ETL/perovskite interface, which can significantly affect the device’s performance. To tackle these limitations, researchers have been exploring alternative strategies, and the adoption of a bilayer ETL has emerged as a promising solution. By integrating the unique properties of both TiO_2_ and SnO_2_ in a double-layer ETL, it becomes possible to overcome the drawbacks associated with a single-layer ETL. The combination of TiO_2_ and SnO_2_ allows for improved electron extraction and transport, as well as the realignment of the band, which contributes to a better charge collection efficiency. Recent studies have demonstrated the successful implementation of TiO_2_/SnO_2_ bilayer ETLs, showcasing remarkable results in enhancing the efficiency of perovskite solar cells. For instance, Song et al. achieved a remarkable power conversion efficiency (PCE) of 21.1% in planar perovskite solar cells using a TiO_2_/SnO_2_ bilayer ETL [35]. Similarly, Tavakoli et al. utilized an amorphous SnO_2_ layer on top of a compact TiO_2_ layer as the ETL, achieving a PCE of 21.4% [36]. Moreover, Li D et al. [37] successfully constructed SnO_2_/TiO_2_ dual ETLs with varying SnO_2_ thicknesses, leading to an outstanding PCE of 21.45%. These impressive results highlight the potential of bilayer ETLs in significantly enhancing the efficiency of perovskite solar cells. In addition to TiO_2_/SnO_2_ bilayer ETLs, researchers have explored other combinations, such as the TiO_2_/WO_3_ bilayer ETL designed by You et al. [38], which efficiently extracts carriers from the perovskite layer to the substrate, resulting in a PCE of 20.14%. Furthermore, Lin et al. [39] reported that a ZnO-modified ITO substrate indirectly contributed to the generation of a large perovskite grain size, leading to an impressive efficiency of 20.45%. However, existing preparation methods often rely on high-temperature processes to improve the crystalline quality of the electron transport layer. Low-temperature preparation methods, on the other hand, mostly involve atomic layer deposition and magnetron sputtering, which can be expensive due to the need for specialized equipment or targets, making them less suitable for large-scale production. In contrast, electron beam evaporation offers the advantage of a lower energy input, which reduces film damage and allows for the use of particle or powder materials in the preparation process. Moreover, by employing evaporative methods for perovskite materials, the entire process of perovskite solar cell fabrication can be carried out under vacuum conditions, facilitating the preparation of tandem and large-area perovskite solar cells.

This paper proposes the fabrication of a SnO_2_/TiO_2_ bilayer electron transport layer (ETL) for planar perovskite solar cells utilizing a low-temperature (200 °C) process. The improved efficiency of the SnO_2_/TiO_2_ bilayer ETL cell can be attributed to the electron transfer from TiO_2_ to SnO_2_ at the interface. This transfer effectively separates electrons and holes in the composite sample, reducing their recombination probability and allowing for the photogenerated electrons to flow to the SnO_2_/TiO_2_ interface, thereby inhibiting the recombination of photogenerated carriers. Additionally, the insertion of a SnO_2_ layer on the surface of TiO_2_ serves to passivate defects, correcting the hysteresis of the perovskite solar cell and improving long-term stability under illumination. As anticipated, the perovskite solar cells incorporating the bilayer ETL exhibit significant improvements in open-circuit voltage (*V*_oc_) and fill factor (FF), indicating the suitability of the bilayer ETL for low-temperature fabrication processes aimed at achieving high-performance perovskite solar cells.

## 2. Materials and Methods

### 2.1. Fabrication of CsPbI_3−x_Br_x_ Perovskite Solar Cells

**Figure 1:**
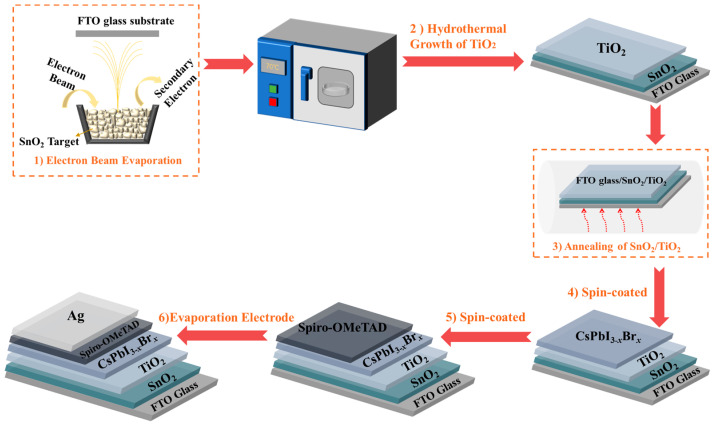
Schematic illustration of the fabrication process for CsPbI_3−x_Br_x_ perovskite solar cell.

As shown in step 1 of Figure 1, SnO_2_ thin films were deposited onto fluorine-doped SnO_2_-coated glass (FTO/glass) using electron beam evaporation. The deposition process involved evacuating the chamber to a base pressure of 5 × 10^−3^ Pa and heating the substrate to 140 °C. SnO_2_ particles served as the evaporation source, with a deposition rate of 0.3 Å·s^−1^ and a film thickness of 30 nm. Oxygen was introduced during deposition while maintaining a pressure of 3.3 × 10^−2^ Pa.

As shown in step 2 of Figure 1, 200 mL of deionized water was poured into a beaker and refrigerated for 12 h until completely frozen. Then, 4.5 mL of TiCl_4_ was slowly added to the ice to melt it, leaving some ice remaining. The FTO glass, on which SnO_2_ had been evaporated, was fixed in a Petri dish using high-temperature adhesive tape. The melted TiCl_4_ was poured into the Petri dish after the ice melted. The Petri dish was then placed in an electric blast drying oven and maintained at 70 °C for 65 min. Afterward, the culture dish was removed and rinsed with deionized water. We used a step profiler to measure the thickness of the TiO_2_ layer prepared using the hydrothermal method, and the thickness was found to be 50 nm.

Step 3, as shown in Figure 1, involved annealing the SnO_2_/TiO_2_ thin film in air at 200 °C for 30 min using an annealing table.

For step 4 of Figure 1, a CsPbI_3−x_Br_x_ solution was prepared by dissolving 380.4 mg HPbI_3_, 187.08 mg CsI, and 73.4 mg PbBr_2_ in DMF and DMSO, followed by filtration with a 0.2 μm syringe filter. CsPbI_3−x_Br_x_ films were cast by spin coating at 4500 r.p.m for 30 s and dried on a hot plate at 200 °C for 5 min.

Step 5 of Figure 1 involved the application of the hole transport layer (HTL) immediately after the perovskite film cooled down. The precursor solution for the HTL was prepared by dissolving 72.3 mg Spiro-OMeTAD, 29 μL TBP, and 18 μL Li-TFSI solution (520 mg Li-TFSI in 1 mL acetonitrile) in 1 mL CB. The HTL was caste by spin coating at 7050 r.p.m. for 30 s in a glove box.

As shown in step 6 of Figure 1, a 70 nm thick Ag layer was deposited on top of the HTL using thermal evaporation through a metal shadow mask. The deposition took place at a base pressure of about 2 × 10^−3^ Pa.

### 2.2. Characterization

The surface morphologies of the samples were analyzed using a Field Emission Scanning Electron Microscope (FESEM, S-4800, Hitachi, Tokyo, Japan) and a scanning probe microscope (Dimension edge, Bruker, Karlsruhe, Germany).

Current density–voltage (*J*−*V*) curves were measured in dry air using a Keithley 2400 source meter under standard 1 sun AM 1.5 simulated solar irradiation system (SAN-EI 100 mw·cm^−2^) from Giant Force Technology Co., Limited. The *J*−*V* measurement was performed at a scan rate of 50 mV·s^−1^.

Photoluminescence (PL) spectra were measured using an Edinburgh FLS 980 fluorescence spectrometer (UK) under excitation at 375 nm. Electrochemical impedance spectroscopy (EIS) measurements were conducted using an electrochemical workstation (PGSTAT 302 N, Autolab, Sursee, Switzerland). X-ray diffraction (XRD) was characterized using a Bruker D8 Discover instrument (Karlsruhe, Germany).

## 3. Results

**Figure 2:**
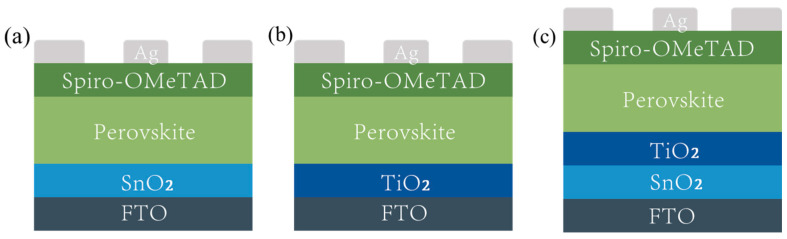
Schematic diagram of device structures of PSCs based on the (**a**) SnO_2_, (**b**) TiO_2_, and (**c**) SnO_2_/TiO_2_.

To investigate the impact of the SnO_2_/TiO_2_ bilayer electron transport layer (ETL) on the performance of perovskite solar cells, the following three types of ETLs were prepared: SnO_2_, TiO_2_, and SnO_2_/TiO_2_. The structure diagram of the perovskite solar cells (PSC) with these ETLs is illustrated in Figure 2a–c.

**Figure 3:**
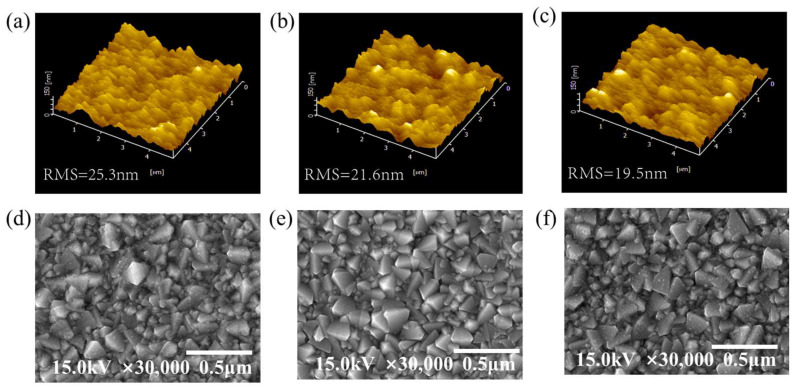
AFM image of (**a**) SnO_2_, (**b**) TiO_2_, and (**c**) SnO_2_/TiO_2_ films. SEM images of the (**d**) SnO_2_, (**e**) TiO_2_, and (**f**) SnO_2_/TiO_2_ filmst.

The atomic force microscopy (AFM) images in Figure 3a–c depict the surface roughness of the three ETLs, with a scan area of 5 × 5 μm^2^. The root-mean-square roughness (RMS) values for the SnO_2_, TiO_2_, and SnO_2_/TiO_2_ films are measured to be 2.53 nm, 2.16 nm, and 1.95 nm, respectively. The SnO_2_/TiO_2_ bilayer exhibits a lower roughness, which is advantageous for perovskite solar cells. The SnO_2_ layer effectively smoothens the roughness of the FTO stack, contributing to improved quality and crystal growth of the perovskite thin films [36].

Furthermore, the top-view scanning electron microscopy (SEM) images in Figure 3d–f illustrate the differences in morphology between the SnO_2_, TiO_2_, and SnO_2_/TiO_2_ thin films. It is evident that the single-layer SnO_2_ and TiO_2_ films exhibit significant pinholes and pores, with small and non-uniform grain sizes. Conversely, the SnO_2_/TiO_2_ bilayer completely covers the FTO substrate, displaying larger and more uniform grain sizes compared to the single-layer films. This uniformity is crucial in reducing current leakage at the interface between the device and the electrode.

**Figure A1:**
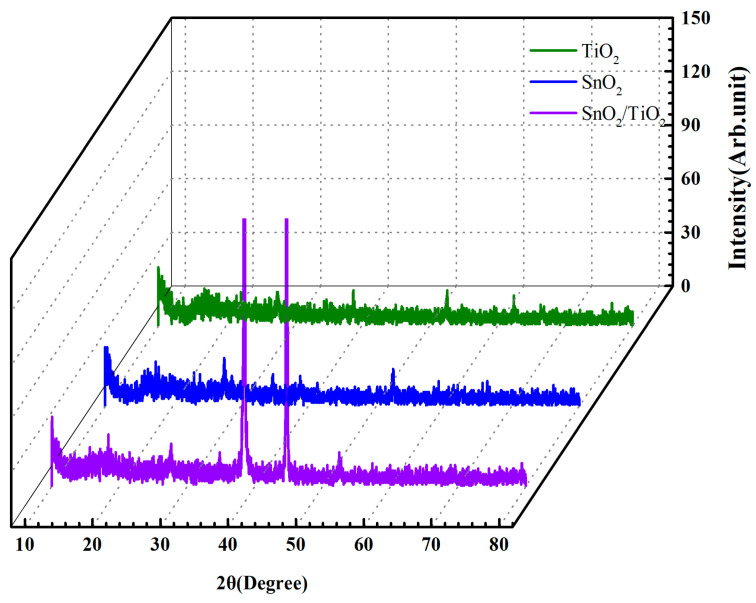
XRD patterns of SnO_2_, TiO_2_, and SnO_2_/TiO_2_ films.

In order to comprehensively assess the quality of the TiO_2_, SnO_2_, and TiO_2_/SnO_2_ layers, X-ray diffraction (XRD) tests were conducted (see Figure A1 in Appendix A for more detailed results). XRD is a potent technique used to analyze the crystal structure and phase composition of materials. The diffraction patterns obtained from the XRD measurements offer valuable information about the arrangement of atoms within the crystal lattice, providing insights into the grain size and crystallinity of the thin films.

The XRD spectra revealed the absence of distinct peaks corresponding to TiO_2_ or SnO_2_ when a single electron transport layer was used, suggesting that the samples may have very small thicknesses of TiO_2_ and SnO_2_ or could be in an amorphous state. However, with the TiO_2_/SnO_2_ bilayer electron transport layer, the XRD diffraction peaks became narrower and stronger. Additionally, slight shifts were observed in comparison to the standard TiO_2_ or SnO_2_ peaks, indicating possible interface interactions or lattice mismatches between the two oxides [40,41]. These factors could influence the band alignment and charge transfer at the interface, necessitating further investigation using complementary techniques like X-ray photoelectron spectroscopy (XPS).

**Figure A2:**
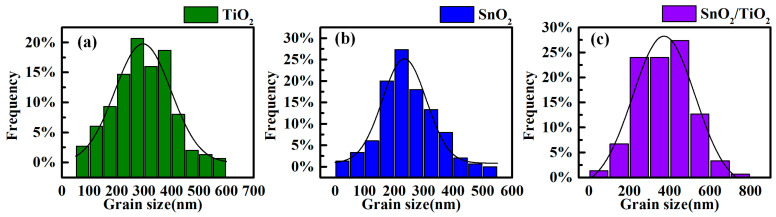
Grain size statistics of (**a**) SnO_2_, (**b**) TiO_2_, and (**c**) SnO_2_/TiO_2_ films.

It is crucial to acknowledge that the XRD peak intensity is influenced by various factors, including structure factors, angle factors, absorption factors, temperature factors, multiplicity factors, and the number of unit cells in a specific crystal plane. The number of unit cells can provide valuable insights into the thickness or relative content of elements in the sample. Thus, the evaluation of the film quality, crystallinity, and grain size in electron transport layers should not solely rely on the XRD peak intensity and width. To supplement the XRD analysis, a statistical examination of the grain size of different electron transport layers was conducted, and the results are presented in Figure A2 in Appendix A.

The statistical analysis revealed that the single-layer SnO_2_ and TiO_2_ films exhibited significant pinholes and pores, indicating smaller and non-uniform grain sizes. In contrast, the SnO_2_/TiO_2_ bilayer, due to its complete coverage of the FTO substrate, exhibited larger and more uniform grain sizes, as well as a smoother surface of the bilayer electron transport layer, which is advantageous for producing high-quality perovskite thin films [41].

The interfacial adhesion of the double-layer SnO_2_/TiO_2_ ETL is another crucial parameter for achieving high device efficiency, as it directly influences the growth quality of the subsequent CsPbI_3−x_Br_x_ films. A lower contact angle indicates higher wettability [42]. Good wettability promotes the spreading and growth of the perovskite solution on the surface, leading to improved perovskite film quality.

**Figure 4:**
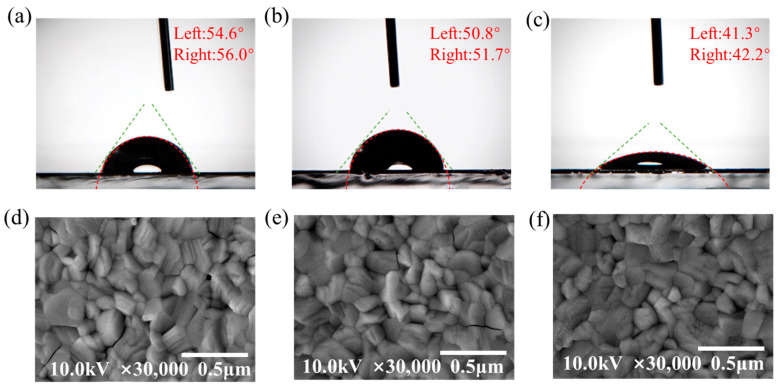
Water contact angle measurement for (**a**) SnO_2_, (**b**) TiO_2_, and (**c**) SnO_2_/TiO_2_ on glass. SEM image of the perovskite film deposited on (**d**) SnO_2_, (**e**) TiO_2_, and (**f**) SnO_2_/TiO_2_ bilayer.

In Figure 4a–c, the contact angles measured on the surfaces of the SnO_2_, TiO_2_, and SnO_2_/TiO_2_ thin films are found to be 55.3°, 51.3°, and 41.8°, respectively. Notably, the SnO_2_/TiO_2_ thin film exhibits the smallest contact angle, indicating a more hydrophilic surface. The high hydrophilicity of the SnO_2_/TiO_2_ film facilitates better spreadability of the polar solvent on the ETL, promoting the formation of a uniform perovskite film. This improved film quality ensures better interface contact between the perovskite layer and the ETL.

**Figure A3:**
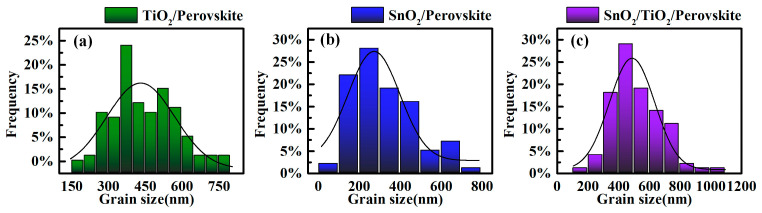
Grain size statistics of the perovskite films spin coated on (**a**) TiO_2_, (**b**) SnO_2_, and (**c**) SnO_2_/TiO_2_ films.

As shown in Figure 4d–f, the top-view SEM images of the perovskite films deposited on the SnO_2_, TiO_2_, and SnO_2_/TiO_2_ electron transport layers (ETLs) exhibit more uniform and larger grain sizes compared to those on the surfaces of the TiO_2_ and SnO_2_ ETLs. To substantiate this observation, we conducted a statistical analysis of the grain size of the perovskite deposited on different ETLs. The statistical results demonstrate that the use of the SnO_2_/TiO_2_ bilayer ETL indeed contributes to enhancing the quality of the perovskite thin film. For detailed results, please refer to Figure A3 in Appendix A. This finding further supports the idea that the SnO_2_/TiO_2_ bilayer ETL facilitates the formation of perovskite films with improved uniformity and larger grain sizes, ultimately leading to enhanced device performance.

In theory, the contact angles of the TiO_2_/SnO_2_ bilayer and the TiO_2_ layer should be similar if the TiO_2_ layer fully covers the SnO_2_ layer. The contact angle measurement is a valuable indicator of the wetting behavior of a liquid on a solid surface, influenced by the surface tension of the liquid, the surface energy of the solid, and the roughness and chemical composition of the solid surface.

Based on the surface roughness analysis of the samples, it was observed that the TiO_2_/SnO_2_ sample exhibited a smaller surface roughness. According to the Wenzel equation, for a hydrophilic solid surface, smaller surface roughness results in a smaller contact angle. We posit that the presence of defects on the FTO surface is significant, and the SnO_2_ film partially smooths the FTO surface, facilitating the formation of a compact perovskite layer. This inference is indirectly supported by the observation of grain stoppage in the perovskite layer. Additionally, the SnO_2_ buffer layer promotes the nucleation and growth of the TiO_2_ layer, leading to an increase in the average grain size. Consequently, the presence of the SnO_2_ film as a suitable buffer layer on the FTO surface can enhance the quality of the TiO_2_ electron transport layer, ultimately improving the solar cell’s performance.

However, it is essential to exercise caution and not oversimplify the relationship between the surface roughness and contact angle. The Wenzel equation is applied when the water droplet completely rests on the surface without any gaps below, known as the Wenzel state. In the Cassie state, where the water droplet is suspended on rough regions with air gaps below, a different set of equations is used to describe the contact angle. In the Cassie state, the contact angle is predominantly influenced by the solid fraction (the amount of solid in contact with the liquid droplet) rather than the surface roughness.

**Figure 5:**
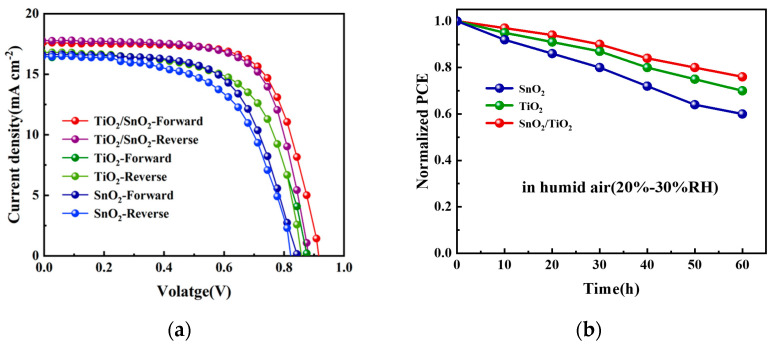
(**a**) Champion *J*–*V* curves of the PSCs with different types of ETLs. (**b**) The device lifetime stability test (20–30% relative humidity for 60 h).

To analyze the photovoltaic performance differences of the different ETLs, 20 perovskite solar cells were prepared under each condition, and their results under illumination were analyzed. Figure 5a presents the forward and reverse scanned *J*–*V* curves of the champion device under the AM1.5 solar simulator. Overall, the stability test results demonstrate that the perovskite solar cells based on the SnO_2_/TiO_2_ bilayer ETL exhibit better stability and stronger electron extraction ability compared to the cells based on the single-layer TiO_2_ ETL and single-layer SnO_2_ ETL. Figure 5b illustrates the results of the humid air storage stability tests for perovskite solar cells based on the SnO_2_/TiO_2_ bilayer ETL, monolayer TiO_2_ ETL, and monolayer SnO_2_ ETL. The cells were subjected to a relative humidity of 20–30% for 60 h. It can be observed that the perovskite solar cells based on the SnO_2_/TiO_2_ bilayer ETLs exhibit better stability in the storage test compared to the cells based on the monolayer TiO_2_ ETL and monolayer SnO_2_ ETL.

The improved stability in the bilayer-ETL-based cells can be attributed to several factors. Firstly, the bilayer ETL contributes to the better film quality of the perovskite layer. The SnO_2_ layer in the bilayer structure helps to smooth the surface roughness of the FTO stack to some extent, leading to an improved film quality of the perovskite layer. Secondly, the bilayer ETL reduces the charge accumulation at the perovskite/ETL interface by enhancing electron extraction. This improved electron extraction capability contributes to the reduction in instability factors and improves device stability.

The statistical data for the *V*_oc_, short circuit current density (*J*_sc_), fill factor (FF), power conversion efficiency (PCE), and hysteresis factor (HF) are summarized in Table 1.

The SnO_2_/TiO_2_ perovskite solar cells exhibit reduced hysteresis compared to the single-layer SnO_2_ or TiO_2_ perovskite solar cells. The hysteresis factor (HF) is calculated based on the three types of perovskite solar cells using the following formula:

$$\mathrm{HF}=\left({\mathrm{PCE}}_{\mathrm{Reverse}}-{\mathrm{PCE}}_{\mathrm{Forward}}\right)÷\;{\mathrm{PCE}}_{\mathrm{Reverse}}$$

The specific values of *V*_oc_, *J*_sc_, FF, PCE, and HI for the different ETLs are listed in Table 1. These data provide insights into the superior performance of the SnO_2_/TiO_2_ bilayer ETL in perovskite solar cells compared to the single-layer SnO_2_ or TiO_2_ counterparts.

The hysteresis factor (HF) of the SnO_2_/TiO_2_ bilayer ETL is smaller than that of the SnO_2_ ETL and TiO_2_ ETL, with the HF of the SnO_2_/TiO_2_ bilayer ETL being the smallest (0.06). This indicates that the bilayer ETL can effectively suppress the hysteresis effect in perovskite solar cells. In perovskite solar cells, hysteresis is mainly caused by charge or ion accumulation and charge transfer imbalance at the ETL/perovskite interface. Reducing the charge accumulation at the perovskite/ETL interface and enhancing electron extraction are effective ways to mitigate the hysteresis effect.

Therefore, the improved electron extraction capability provided by the bilayer ETL is attributed to the mitigation of the hysteresis effect. By facilitating efficient electron extraction from the perovskite layer, the bilayer ETL helps to reduce charge accumulation and charge transfer imbalances, resulting in improved device performance and reduced hysteresis in perovskite solar cells.

**Figure 6:**
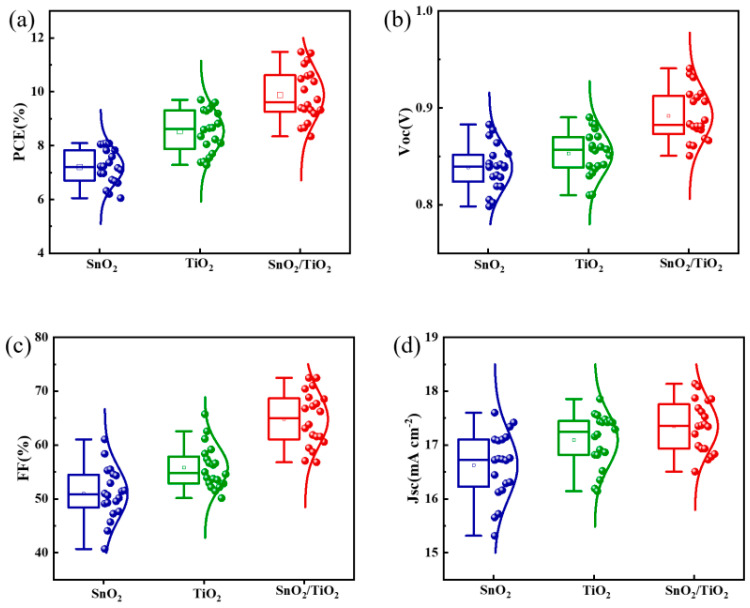
The box charts of the PSCs with the different ETLs (**a**–**d**); the number of total samples accounted for statistics is 20.

The winner device based on the SnO_2_/TiO_2_ bilayer ETL achieved a PCE of 11.48%, *V*_oc_ of 0.91 V, *J*_sc_ of 17.68 mA cm^−2^, and FF of 71.68%. In comparison, the PCEs of the single-layer SnO_2_ and TiO_2_ devices were 8.09% and 9.60%, respectively. The average fill factor of the bilayer electron transport layer is approximately 15% higher compared to the single-layer electron transport layer. The box plots in Figure 6a–d demonstrate the PCE distribution and additional photovoltaic parameters for the *J*–*V* measurements for all three types of ETLs (total number of samples: 20 devices). This indicates that the devices exhibit high repeatability.

The SnO_2_/TiO_2_ perovskite solar cells exhibit superior performance compared to the single-layer SnO_2_ and TiO_2_ devices. The *V*_oc_ and FF parameters of the SnO_2_/TiO_2_ bilayer ETL are significantly higher than those of the single-layer SnO_2_ ETL, as shown in Figure 6b,c. This improvement can be attributed to the smoother surface roughness of the bilayer ETL, the higher energy level of the conduction band minimum (ECBM) of the TiO_2_ layer, and the enhanced film quality of the perovskite layer. Notably, the highest *V*_oc_ value achieved by the SnO_2_/TiO_2_ perovskite solar cell is 0.91 V, with an average *V*_oc_ of 0.89 V. This result demonstrates that the low-temperature fabrication of the bilayer ETL is an effective method for improving *V*_oc_. The improved device performance is believed to be a result of good electron extraction and reduced recombination at the interface between the perovskite layer and the bilayer ETL [43].

The conduction band of electron-beam-evaporated SnO_2_ thin films typically ranges from −4.06 eV to −4.21 eV relative to the vacuum, while the valence band levels are between −8.56 eV and −8.12 eV [41,44,45,46]. In contrast, the TiO_2_ prepared via the hydrothermal method has a conduction band around −3.93 eV and a valence band around −7.59 eV, respectively [37].

**Figure 7:**
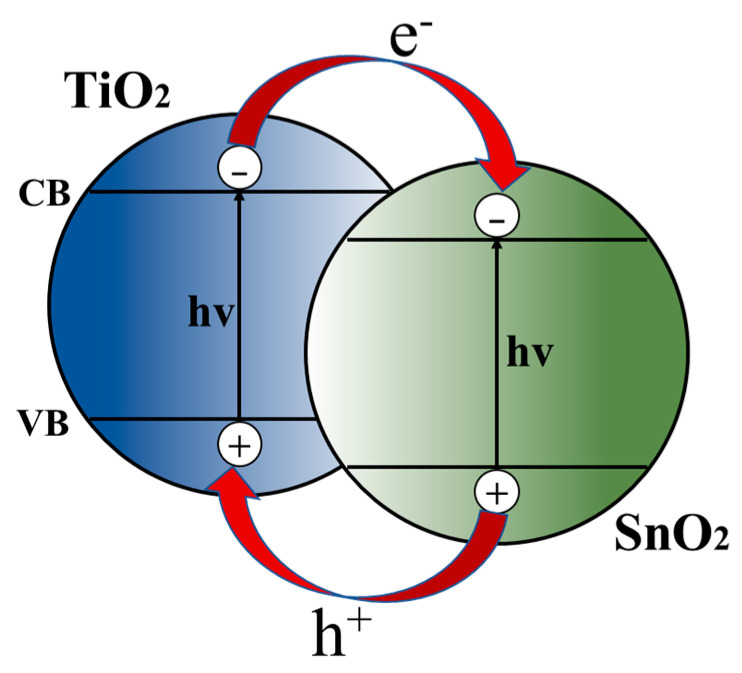
Charge separation mechanism of SnO_2/_TiO_2_ bilayer composite films.

Upon forming the SnO_2_/TiO_2_ bilayer electron transport layer (ETL), the energy level arrangement facilitates electron migration from the conduction band of the TiO_2_ to the conduction band of the SnO_2_ and facilitates hole migration from the valence band of the SnO_2_ to the valence band of the TiO_2_, as illustrated in Figure 7. Consequently, this energy level alignment in the composite film promotes the effective separation of photogenerated electrons and holes, thereby reducing the likelihood of recombination.

**Figure A4:**
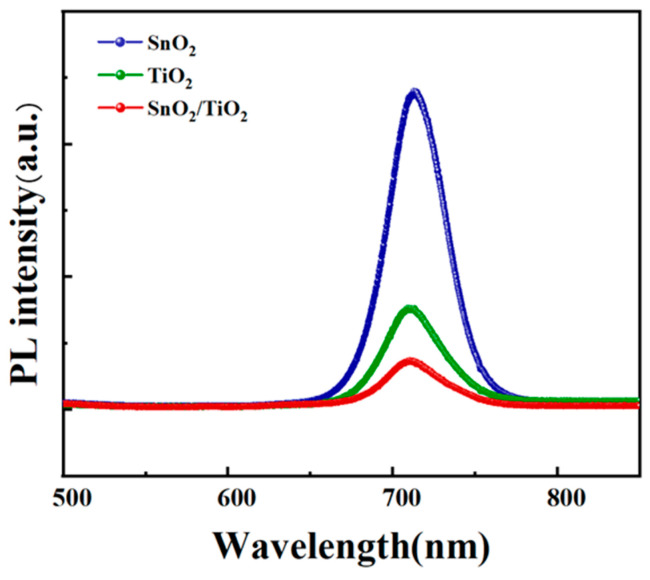
Photoluminescence (PL) spectra of perovskite film deposited on different ETLs.

To further investigate the mechanism behind the enhanced performance of perovskite solar cells (PSCs) with the SnO_2_/TiO_2_ bilayer ETL, we included the photoluminescence (PL) intensity spectra of the perovskite thin films deposited on the three different ETLs. Excitation was achieved using a 375 nm laser on the FTO side. Detailed results can be found in Figure A4 in Appendix A. Notably, the perovskite films on the SnO_2_/TiO_2_ bilayers display a stronger quenching effect compared to the single-layer SnO_2_ and TiO_2_ films. This observation indicates that the SnO_2_/TiO_2_ bilayers effectively reduce the interface defects between the ETL and the perovskite, enabling more efficient charge transfer from the perovskite to the SnO_2_/TiO_2_ bilayers [40].

**Figure 8:**
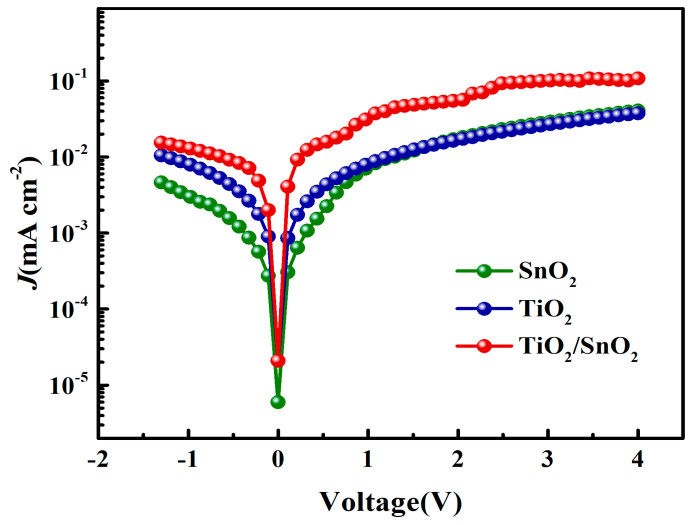
The semi-logarithmic plots of the dark *J*–*V* curves.

To further investigate the charge transfer and recombination in the perovskite solar cells (PSCs), we conducted dark *J*–*V* testing, as depicted in Figure 8. The rectification characteristics unveiled that the SnO_2_/TiO_2_ bilayer demonstrated a higher current density owing to the excellent conductivity of SnO_2_, which occupied defect sites and suppressed surface traps on the TiO_2_ layer, thus facilitating efficient electron extraction from the perovskite absorber. Moreover, the high mobility of the SnO_2_ interfacial layer played a pivotal role in maintaining charge balance within the PSC [47].

**Figure A5:**
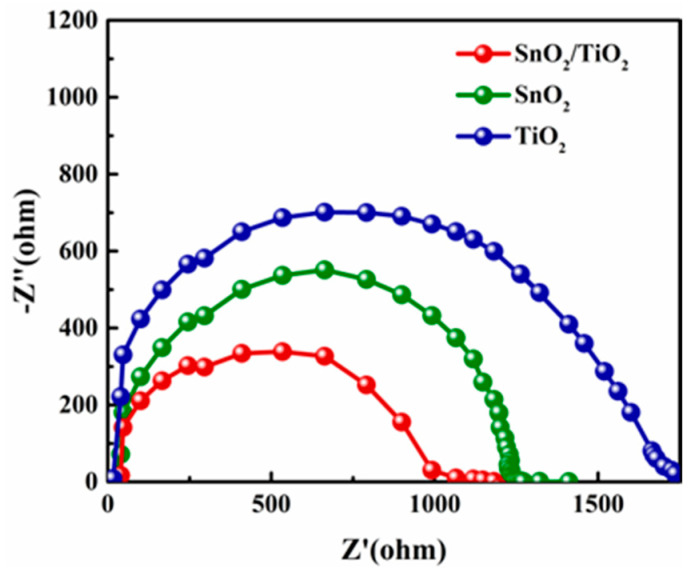
Nyquist plots for the PSCs with different ETLs.

We performed electrochemical impedance spectroscopy (EIS) testing on the samples, with the PSCs tested in a frequency range between 1 MHz and 10 Hz. Detailed results are presented in Figure A5 in Appendix A. In the Nyquist plots, the SnO_2_/TiO_2_ bilayer ETL shows the smallest semicircle, indicating its superior electron extraction ability [37]. Since the primary function of the electron transport layer is to transfer photogenerated electrons from the perovskite layer to the electrode, a lower charge transfer resistance signifies a faster electron transfer from the perovskite layer to the electrode, resulting in a smoother and more efficient electron extraction. Consequently, the SnO_2_/TiO_2_ bilayer ETL indeed exhibits a stronger electron extraction ability, which benefits photogenerated carrier separation and transport.

## 4. Conclusions

In conclusion, our study presents a low-temperature process utilizing electron beam evaporation to fabricate SnO_2_/TiO_2_ bilayer ETL efficient perovskite solar cells with excellent electrical properties. The characterization results confirm the effectiveness of this strategy. The bilayer structure improves the electron extraction ability, as evidenced by the dark-state *J*–*V* measurements. The perovskite solar cell based on the SnO_2_/TiO_2_ bilayer ETL achieves a champion PCE of 11.48% and exhibits better stability in the humidity test compared to the single-layer TiO_2_ and SnO_2_ ETLs. The average fill factor of the bilayer electron transport layer is approximately 15% higher compared to the single-layer electron transport layer. These results demonstrate the potential of the low-temperature annealed SnO_2_/TiO_2_ bilayer ETL films prepared using electron beam evaporation as substitutes for traditional high-temperature sintering ETLs. This technique holds promise for large-area fabrication and commercialization of high-efficiency flexible perovskite solar cells.

## Figures and Tables

**Table 1:** Summary of photovoltaic performance of the PSCs incorporating different ETLs (20 PSCs).

Samples	Scan Direction	*V*_oc_ (V)	*J*_sc_ (mA cm^−2^)	FF (%)	PCE (%)	HF
	Forward	0.82	16.72	49.11	6.73	
SnO_2_	Reverse	0.85	16.74	56.67	8.09	0.17
	Average	0.84 ± 0.02	16.62 ± 0.60	51.01 ± 4.68	7.21 ± 0.63	
	Forward	0.86	17.58	57.07	8.64	
TiO_2_	Reverse	0.87	16.78	65.73	9.60	0.10
	Average	0.85 ± 0.02	17.09 ± 0.48	55.82 ± 3.90	8.54 ± 0.78	
	Forward	0.88	17.87	68.53	10.82	
SnO_2_/TiO_2_	Reverse	0.91	17.68	71.68	11.48	0.06
	Average	0.89 ± 0.03	17.35 ± 0.46	64.80 ± 4.89	9.88 ± 0.95	

## Data Availability

Data sharing does not apply to this article.
